# Supplementary material for: Mice carrying nonsense mutant p53 develop frequent multicentric or metastatic tumors
Source: Cell Death Dis. 2025 Dec 11;17(1):85. doi: 10.1038/s41419-025-08290-9 (PMC12830816; doi:10.1038/s41419-025-08290-9)
Supplement: Supplementary file 5 — Supplementary Figure S1 [file 41419_2025_8290_MOESM5_ESM.pdf]

Supplementary Figure S1

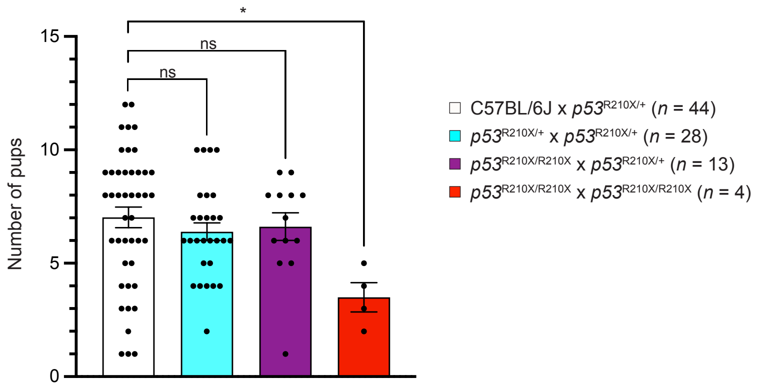

Supplementary Figure S1. Intercrosses of *Trp53*$^{\text{R210X/R210X}}$ mice yield significantly fewer pups, related to Supplementary Table S1.

Graph showing number of pups in each litter born from different intercrosses of *Trp53*$^{\text{R210X}}$ mice. Intercrosses involving *Trp53*$^{\text{R210X/+}}$ mice show an average litter size comparable to that of WT backcross breedings, whereas intercrosses of only *Trp53*$^{\text{R210X/R210X}}$ mice result in significantly smaller litters, *$p < 0.05$. Comparisons to backcross breeding with C57BL/6J WT mice were performed using one-way ANOVA followed by Dunnett's multiple comparison test. Bar heights indicate average number of pups/litter for the different crosses; number of litters for each intercross is indicated. SEM is shown.
