# Supplementary material for: Mice carrying nonsense mutant p53 develop frequent multicentric or metastatic tumors
Source: Cell Death Dis. 2025 Dec 11;17(1):85. doi: 10.1038/s41419-025-08290-9 (PMC12830816; doi:10.1038/s41419-025-08290-9)
Supplement: Supplementary file 6 — Supplementary Figure S2 [file 41419_2025_8290_MOESM6_ESM.pdf]

Supplementary Figure S2A

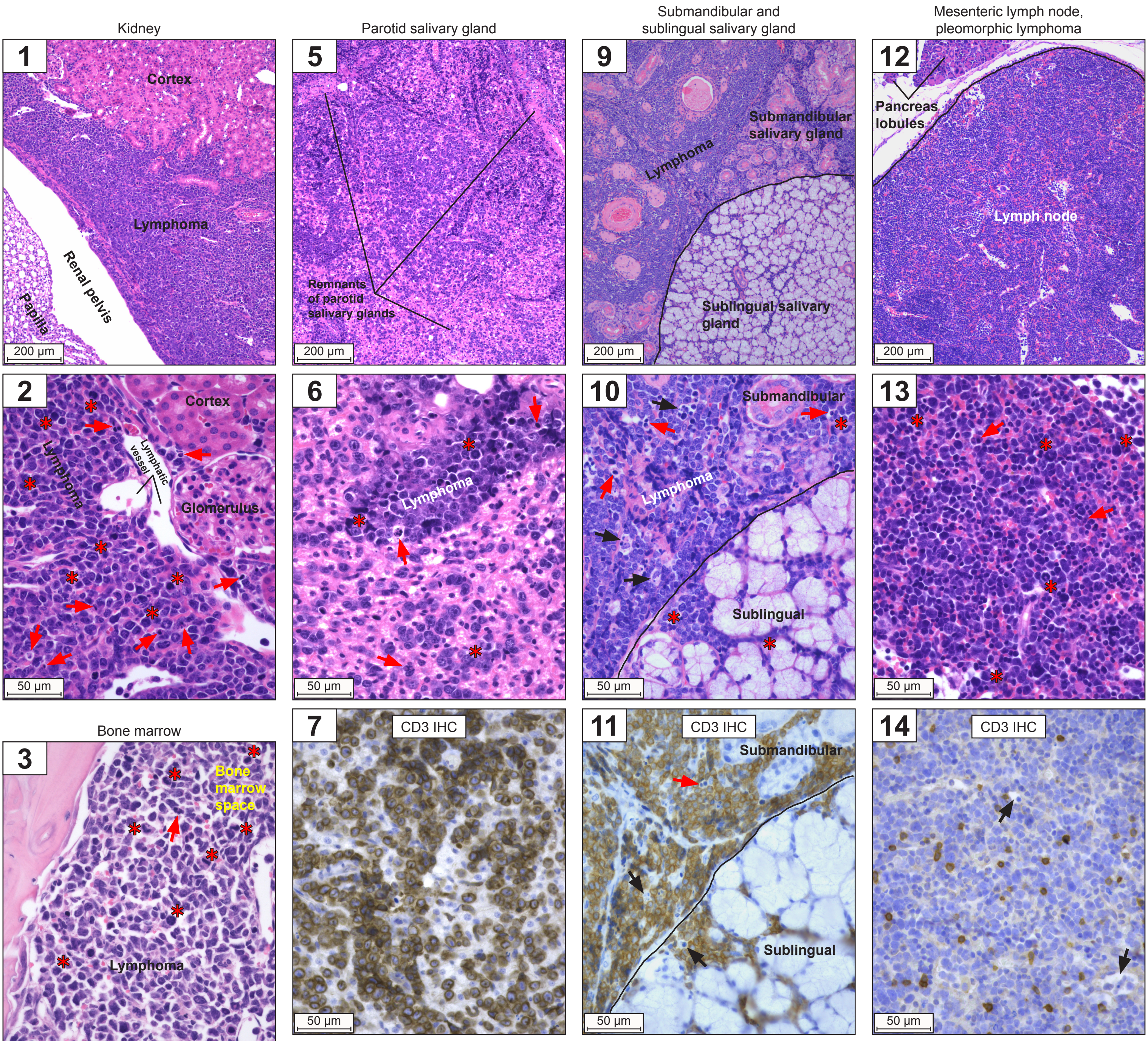

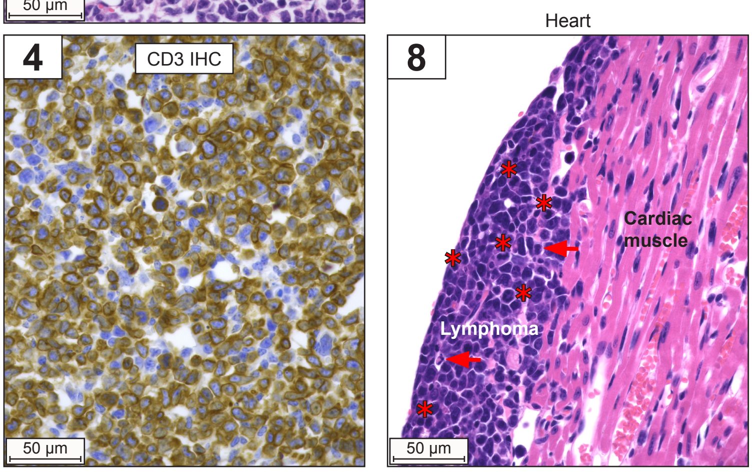

Supplementary Figure S2A. Histomicrographs of T-cell lymphomas and pleomorphic lymphoma in kidney, bone marrow, heart, salivary glands and mesenteric lymph node from *Trp53*$^{R210X/R210X}$ mice, related to Figure 3.
Representative histomicrographs of lymphomas in different organs. Panel 1-11: T-cell lymphomas in kidney (1-2), bone marrow (3-4), parotid salivary gland (5-7), heart (8), submandibular and sublingual salivary gland (9-11). Panel 12-14: Pleomorphic lymphoma in mesenteric lymph node. Red asterisk: mitotic figure; red arrow: single-cell death; black arrow: tingible body machrophage. Black line in panels 9-12 delineates the expected outline of the organ indicated. Panel 1-3, 5-6, 8-10 and 12-13: hematoxylin-eosin staining. Immunohistochemical (IHC) staining in panel 4, 7, 11 and 14 shows CD3-positive cells with moderate to strong cytoplasmic and membrane staining. Note the "starry sky" effect of the numerous tingible body macrophages indicated by black arrows in panel 14. Scale bars: 200µm and 50µm as indicated.

Supplementary Figure S2B

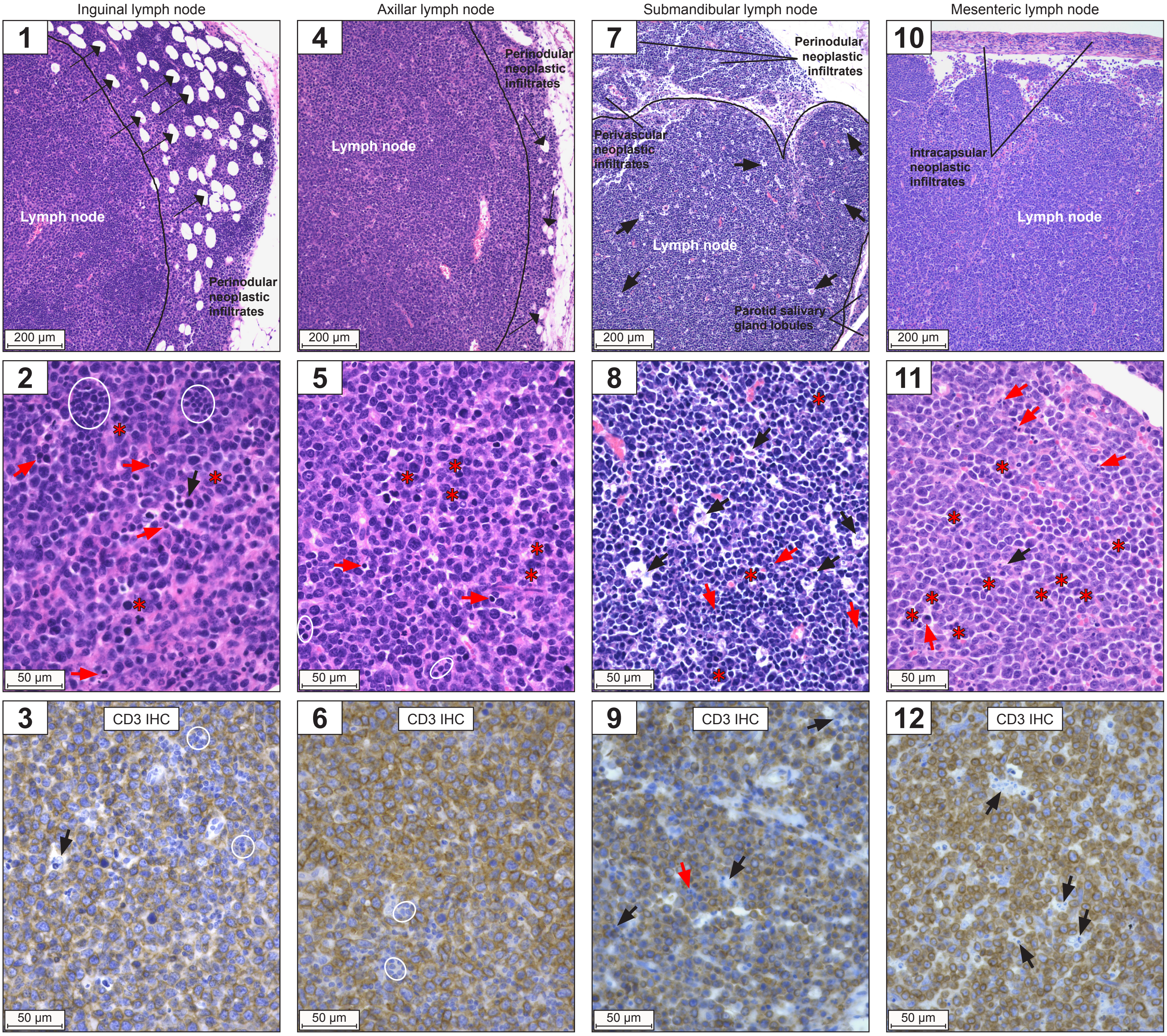

Supplementary Figure S2B. Histomicrographs of T-cell lymphomas in inguinal, axillar, submandibular and mesenteric lymph nodes from *Trp53*$^{R210X/R210X}$ mice, related to Figure 3.

Representative histomicrographs of T-cell lymphomas in inguinal (panels 1-3), axillar (4-6), submandibular (7-9) and mesenteric (10-12) lymph nodes from *Trp53*$^{R210X/R210X}$ mice. Red asterisk: mitotic figure; red arrow: single-cell death; black thick arrow: tingible body machrophage; black thin arrow: lipocytes in perinodal connective tissue. Black line in panels 1, 4 and 7 indicates approximate outline of lymph node. Upper and middle rows: hematoxylin-eosin staining. Aggregates of small, mature CD3 negative lymphocytes, i.e. remnants of the normal lymphocyte population in the lymph node, are circled. Note the ”starry sky” effect of the numerous tingible body macrophages indicated by black arrows in submandibular lymph node. Scale bars: 200µm (upper row) and 50µm (middle and bottom rows) as indicated.

Supplementary Figure S2C

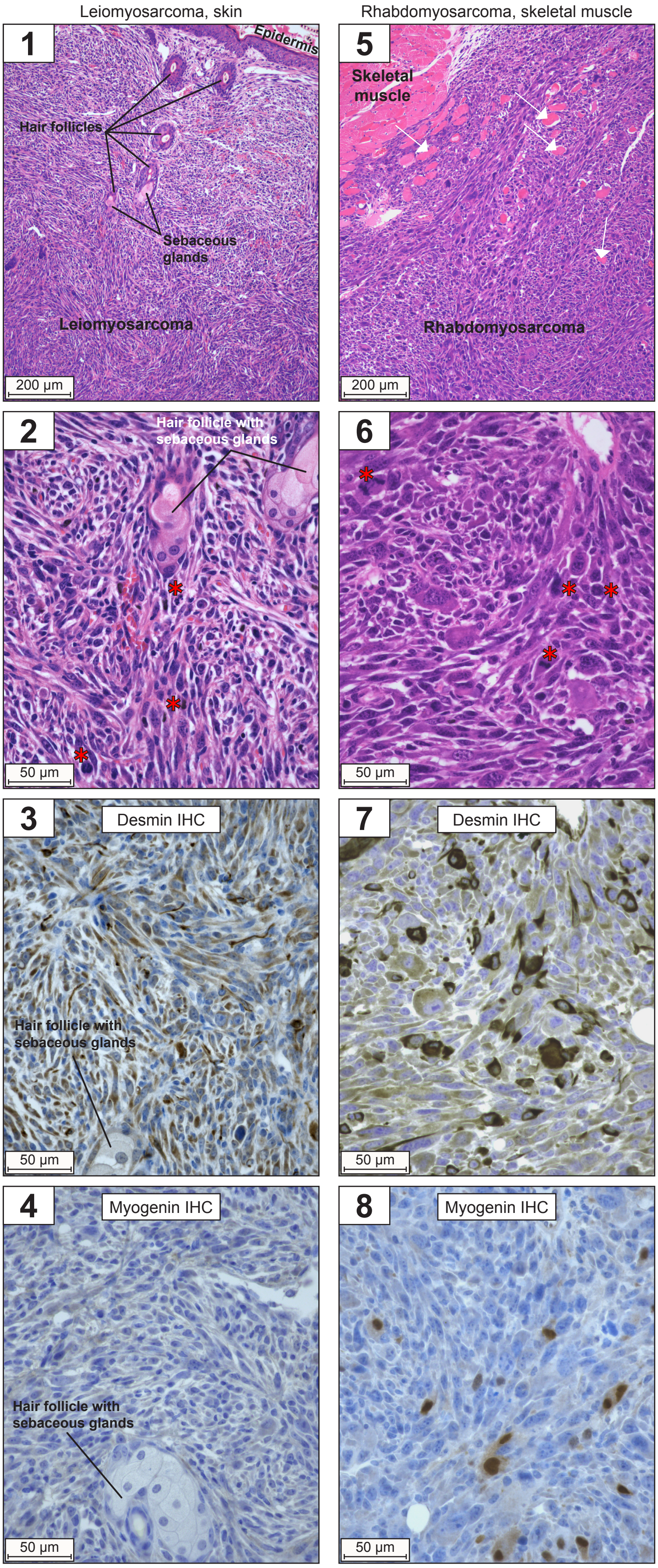

Supplementary Figure S2C. Histomicrographs of leiomyosarcoma and rhabdomyosarcoma in skin and skeletal muscle, respectively, from *Trp53*$^{R210X/R210X}$ mice, related to Figure 3.
Representative histomicrographs of leiomyosarcoma and rhabdomyosarcoma from *Trp53*$^{R210X/R210X}$ mice. Panel 1-2 and 5-6: hematoxylin-eosin staining. Red asterisk: mitotic figure; white thin arrow: entrapped well-differentiated myocoytes, found in the infiltrative borders of the tumor (panel 5). Panel 3 and 7: strong to moderate cytoplasmic desmin staining verifies myocyte origin. Panel 4: no staining for myogenin, consistent with leiomyocyte origin. Panel 8: dispersed neoplastic cells with strong nuclear myogenin staining verifies rhabdomyocyte origin. Scale bars: 200μm (upper row) and 50μm as indicated.
