# Supplementary material for: Mice carrying nonsense mutant p53 develop frequent multicentric or metastatic tumors
Source: Cell Death Dis. 2025 Dec 11;17(1):85. doi: 10.1038/s41419-025-08290-9 (PMC12830816; doi:10.1038/s41419-025-08290-9)
Supplement: Supplementary file 7 — Supplementary Figure S3 [file 41419_2025_8290_MOESM7_ESM.pdf]

Supplementary Figure S3A

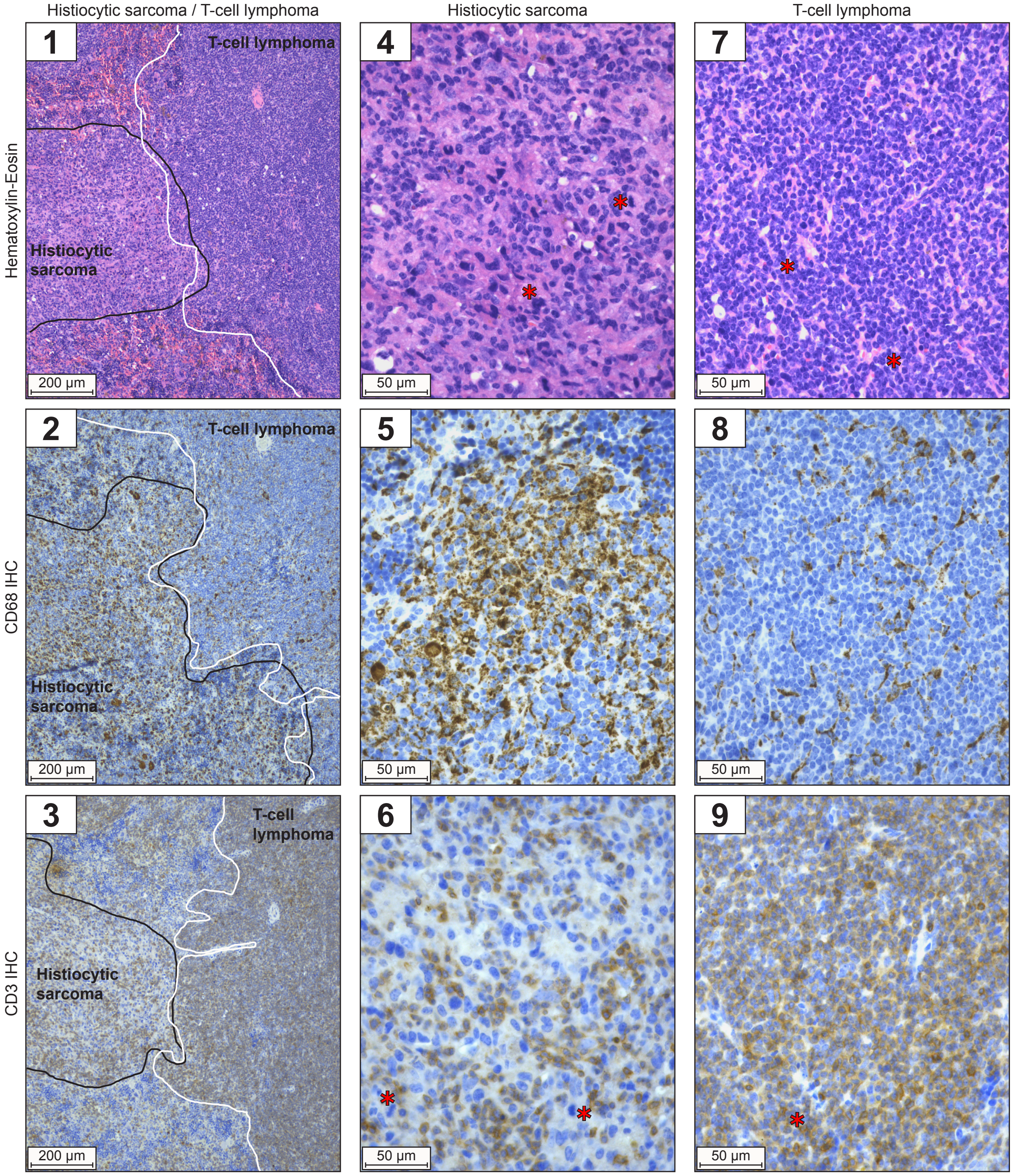

Supplementary Figure S3A. Histomicrographs of histiocytic sarcoma and T-cell lymphoma in spleen from *Trp53*$^{R210X/+}$ mice, related to Figure 4.

Panel 1-3: Spleen with histiocytic sarcoma (outlined by black line, left side of each panel) and T-cell lymphoma (outlined by white line, right side of each panel). Histiocytic sarcoma with numerous CD68-positive cells (moderate to strong cytoplasmic and membrane staining), i.e. histiocytes that form infiltrating sheets, with dispersed CD3-positive T-lymphocytes (strong cytoplasmic staining). T-cell lymphoma with numerous CD3-positive cells (moderate to strong cytoplasmic and membrane staining), i.e. T-lymphocytes that form expansive sheets, with few dispersed CD68-positive histiocytes (strong cytoplasmic staining). Scale bars: 200µm.

Panel 4-6: Histiocytic sarcoma with numerous neoplastic CD68-positive cells (moderate to strong cytoplasmic staining) with pleomorphic appearance and dispersed CD3-positive cells (moderate to strong cytoplasmic and membrane staining), i.e. T-lymphocytes.

Panel 7-9: T-cell lymphoma with numerous CD3-positive cells (moderate to strong cytoplasmic and membrane staining), i.e. T-lymphocytes, and few dispersed CD68-positive cells (strong cytoplasmic staining) with uniform appearance.

Note mitotic figures (red asterisks) in CD3-positive cells in the lymphoma and in CD3-negative cells in the histiocytic sarcoma. Scale bars: 50µm

Supplementary Figure S3B

Histomicrographs of leiomyosarcoma in preputial gland and seminal vesicles, and rhabdomyosarcoma in cheek skin from *Trp53*$^{R210X/+}$ mice, related to Figure 4.

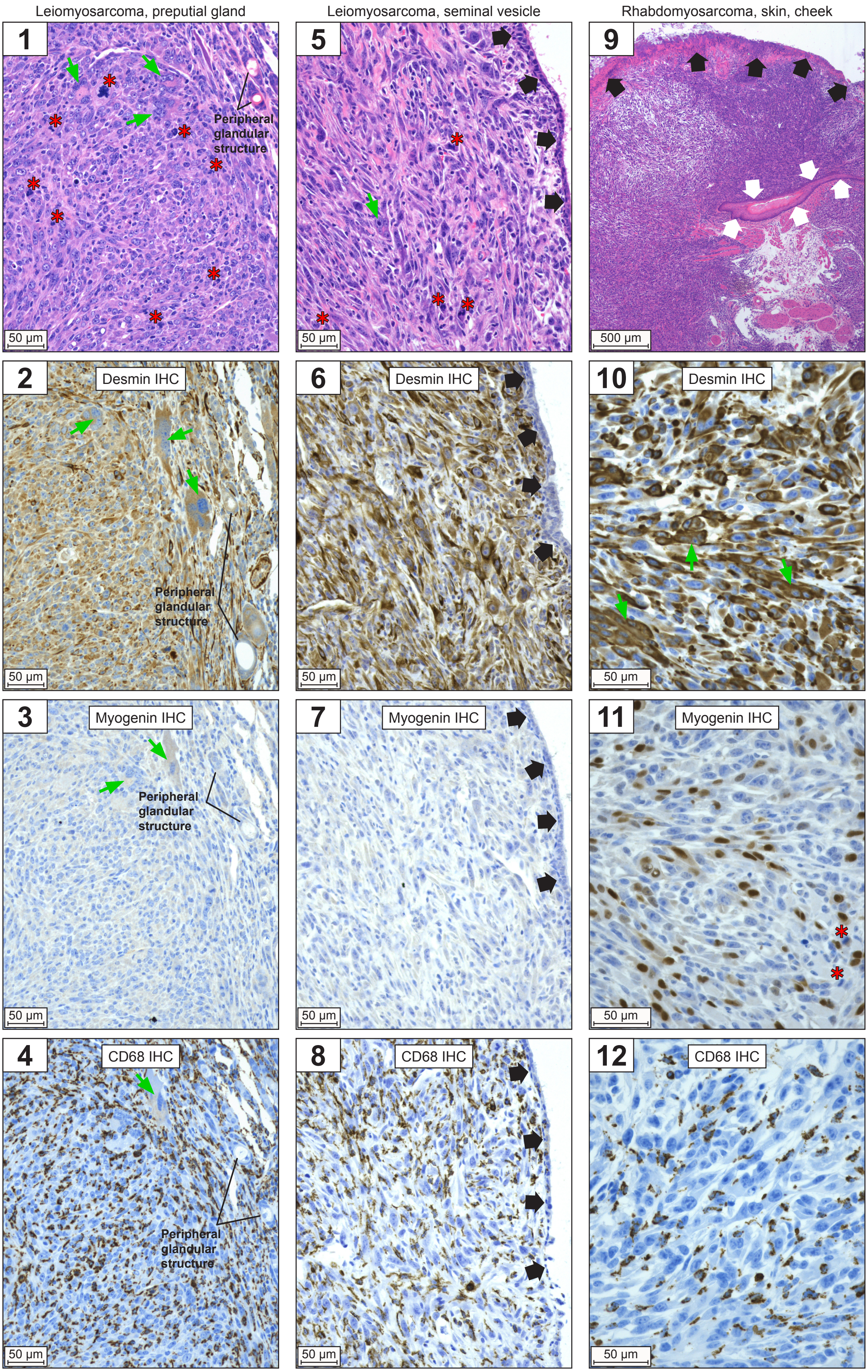

Leiomyosarcoma. Panel 1-4: Preputial gland, almost effaced by a dense proliferation of neoplastic mesenchymal cells. Only few peripheral glandular structures remain.

Panel 5-8: Seminal vesicle lamina propria is severely expanded and effaced by a dense proliferation of neoplastic mesenchymal cells. The epithelial lining is mostly intact (short broad black arrows).

The neoplastic cells in panels 1-8 are spindle-shaped to polygonal and form broad interlacing bundles in a sparse stroma. The cells are highly pleomorphic, with giant multinucleated cells (green arrows) and mitotic figures (red asterisks). Panel 1 and 5: hematoxylin-eosin staining. IHC confirms leiomyocyte origin: all neoplastic cells have moderate to strong cytoplasmic desmin staining, negative nuclear myogenin staining and negative CD68 staining. CD68-positive cells are dispersed, uniform histiocytes infiltrating the tumor. Scale bars: 50µm.

Rhabdomyosarcoma (skin, cheek). Panel 9-12: The epidermis is ulcerated (broad black arrows) with an intact epidermal fold in part of the section (broad white arrows). The dermis and subcutis is severely expanded and effaced by a dense proliferation of neoplastic mesenchymal cells. The cells are highly pleomorphic, with elongated multinucleated cells with the nuclei lining up, i.e. ”strap-cells” (green arrows), and mitotic figures (red asterisks). Panel 9: hematoxylin-eosin staining. IHC confirms rhabdomyocyte origin: all neoplastic cells have moderate to strong cytoplasmic desmin staining, some also have strong nuclear myogenin staining, and all have negative CD68 staining. CD68-positive cells are dispersed, uniform histiocytes infiltrating the tumor. Scale bars: 500µm (panel 9) and 50µm (panel 10-12) as indicated.

Supplementary Figure S3C

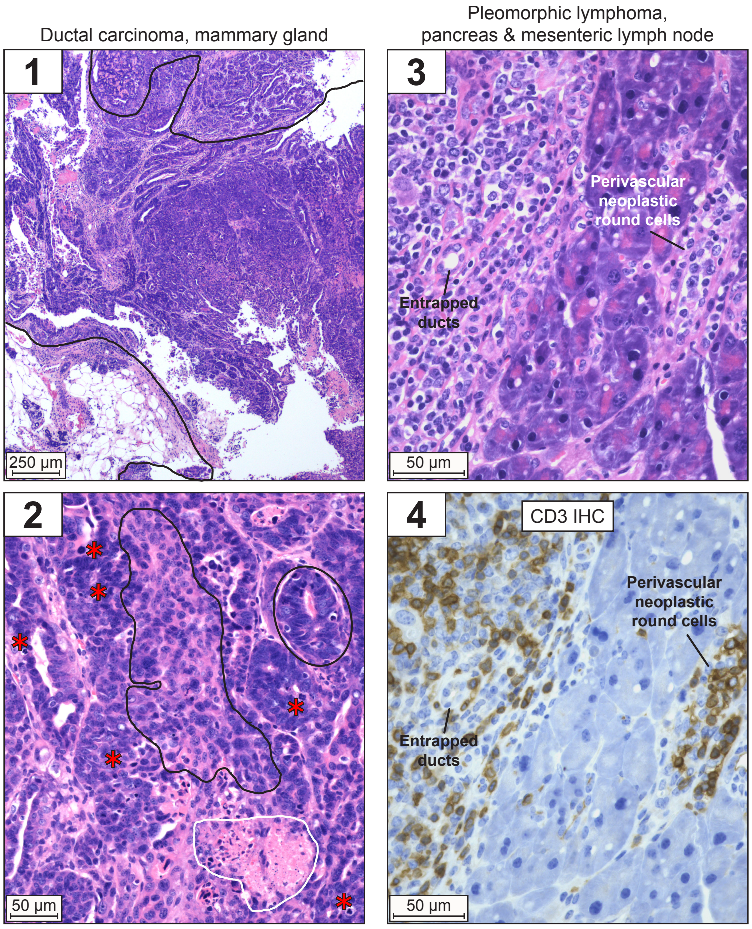

Supplementary Figure S3C. Histomicrographs of ductal carcinoma and pleomorphic lymphoma in mammary gland and pancreas, respectively, from *Trp53*$^{R210X/+}$ mice, related to Figure 4.

Ductal carcinoma in mammary gland. Panel 1 shows a malignant epithelial tumor (between black lines) with normal mammary gland tissue in the lower part of the image, and with hyperplastic mammary gland tissue in the upper part. Panel 2 shows dense proliferations of neoplastic epithelial cells that form ductular (circled) and solid proliferations (black solid outline) in a sparse stroma with multifocal necroses (white solid outline). Red asterisks: mitotic figures.

Pleomorphic lymphoma in pancreas and mesenteric lymph node. Panel 3 shows that the pancreas is multifocally effaced by neoplastic round cells (left side of the image) which are also found perivascularly in the more intact parts of the pancreas. In the effaced area, remaining entrapped ducts can be seen. Panel 4 shows that about half, or less than half, of the neoplastic round cells are CD3-positive.

Panel 1-3: hematoxylin-eosin staining. Scale bars: 250µm and 50µm as indicated.
