# Supplementary material for: Mice carrying nonsense mutant p53 develop frequent multicentric or metastatic tumors
Source: Cell Death Dis. 2025 Dec 11;17(1):85. doi: 10.1038/s41419-025-08290-9 (PMC12830816; doi:10.1038/s41419-025-08290-9)
Supplement: Supplementary file 8 — Supplementary Figure S4 [file 41419_2025_8290_MOESM8_ESM.pdf]

Supplementary Figure S4A

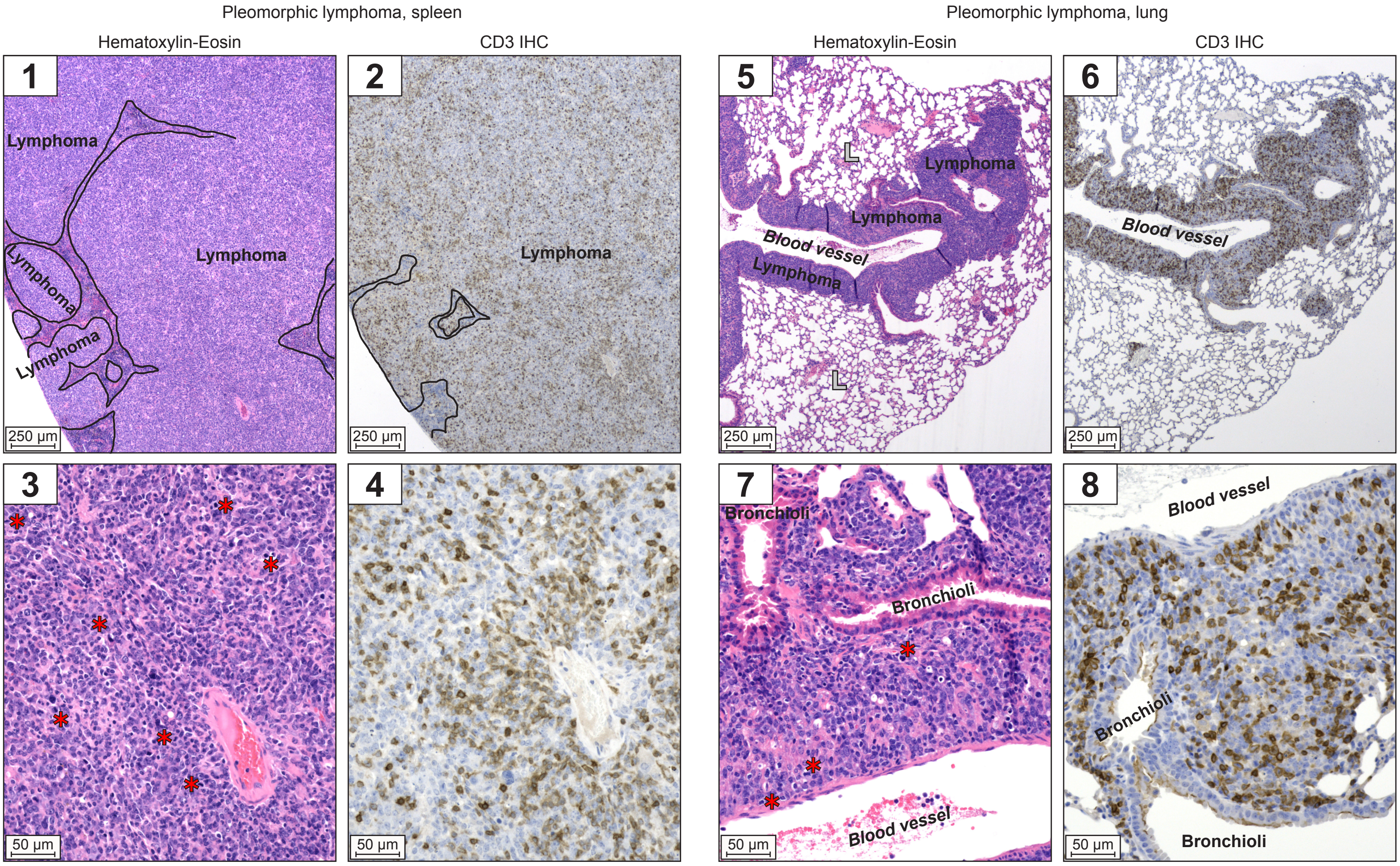

Supplementary Figure S4A. Histomicrographs of pleomorphic lymphoma in spleen and lung from WT mice, related to Figure 5A.

<u>Pleomorphic lymphoma in spleen.</u> Panels 1-4 show dense multinodular to coalescing infiltrates (outlined in black) of neoplastic lymphocytes, a subpopulation of which are strongly CD3-positive, that efface almost all of the normal spleen architecture, and diffusely infiltrate the remaining parts. Red asterisks: mitotic figures.

<u>Pleomorphic lymphoma in lung.</u> Panels 5-8 show dense perivascular infiltrates of neoplastic lymphocytes, a subpopulation of which are strongly CD3-positive. Red asterisks: mitotic figures; L: normal lung parenchyma.

Scale bars: 250µm and 50µm as indicated.

Supplementary Figure S4B

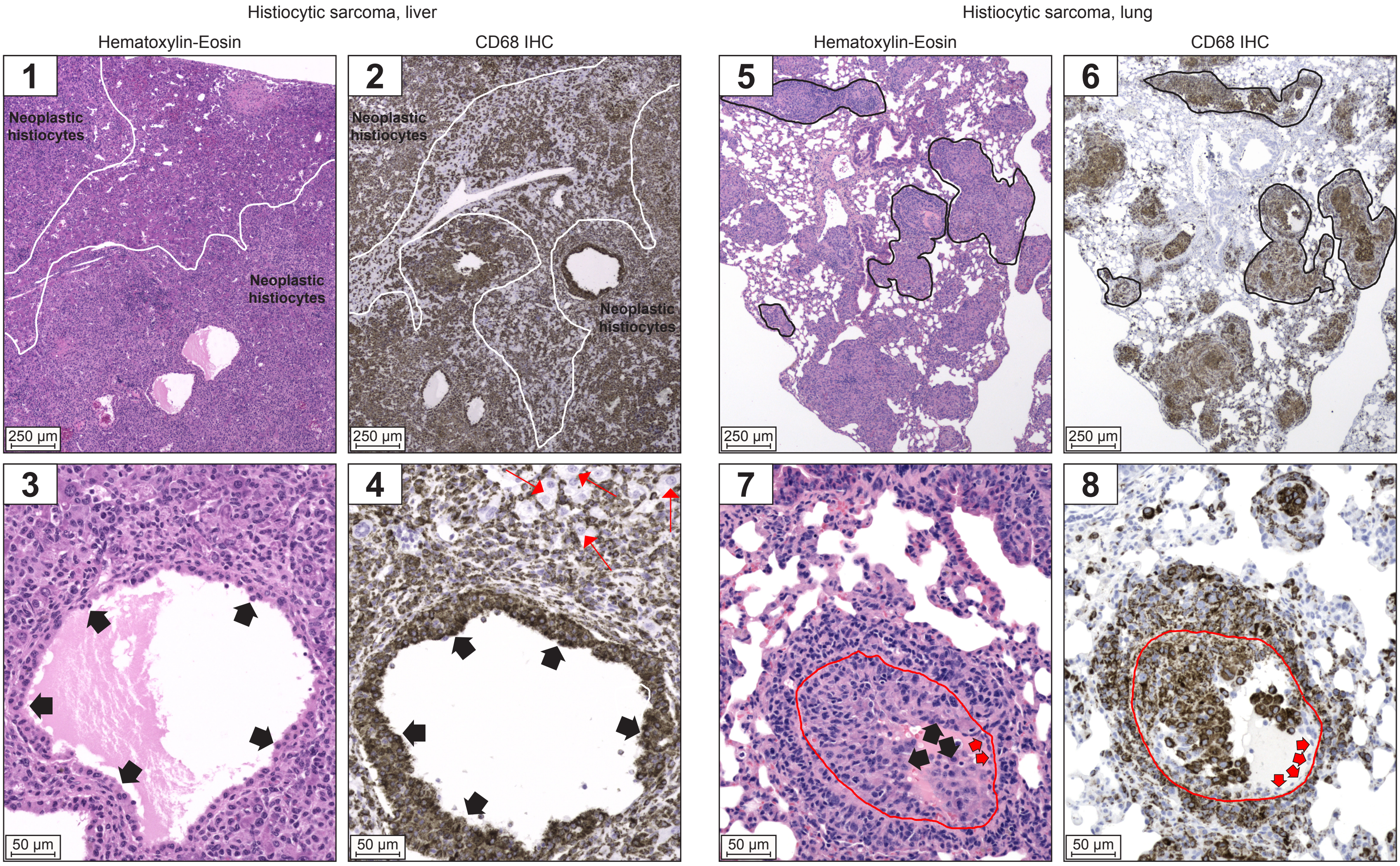

Supplementary Figure S4B. Histomicrographs of histiocytic sarcoma in liver and lung from WT mice, related to Figure 5A.

<u>Histiocytic sarcoma in liver.</u> Panels 1-2 show dense infiltrates of neoplastic histiocytes (outlined by white solid line) efface large parts of the liver, and diffusely infiltrate the remaining parts. Panels 3-4 show a very dilated blood vessel surrounded by dense mats of neoplastic histiocytes. Neoplastic histiocytes are piling up along the endothelium (short broad black arrows) and efface the vessel wall. There are few remaining viable hepatocytes (red thin arrows) among the pleomorphic strongly CD68-positive neoplastic cells.

<u>Histiocytic sarcoma in lung.</u> Panels 5-6 show multinodular to coalescing, vessel centered, dense infiltrates of pleomorphic, strongly CD68-positive neoplastic histiocytes (examples outlined in black solid line) efface large parts of the lung. Panels 7-8 show a very dilated blood vessel surrounded by dense mats of neoplastic histiocytes. Neoplastic histiocytes are piling up along the endothelium (short broad black arrows) and efface the vessel wall. Only small rests of normal, flat endothelium (short broad red arrows) remain. Red line demarcates the approximate location of the endothelial basal lamina.

Scale bars: 250µm and 50µm as indicated.

Supplementary Figure S4C

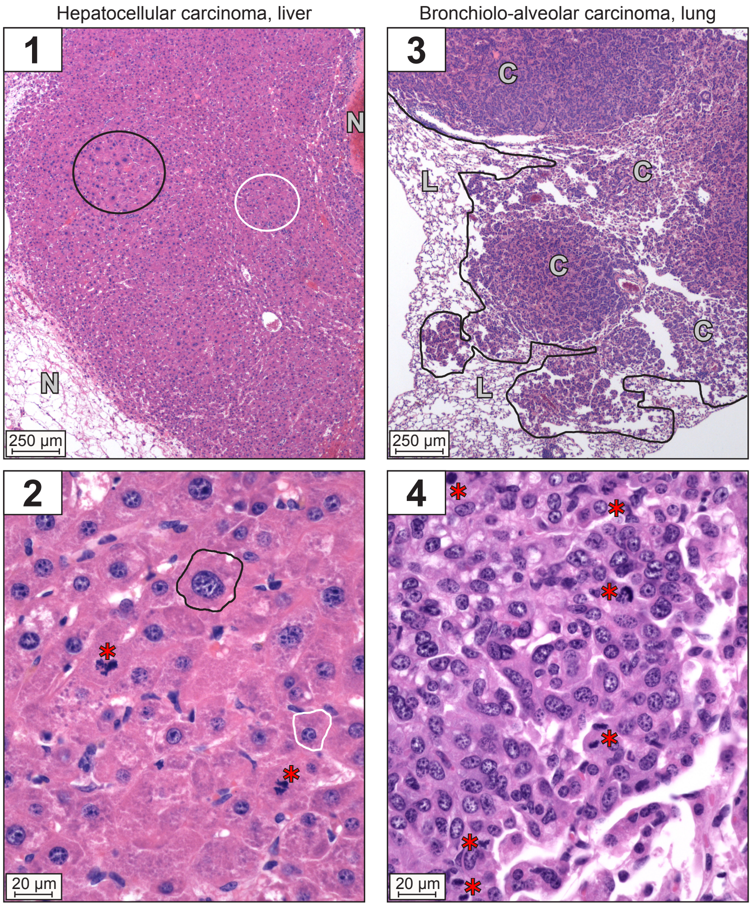

Supplementary Figure S4C. Histomicrographs of hepatocellular and bronchiolo-alveolar carcinoma in liver and lung, respectively, from WT mice, related to Figure 5A.

Hepatocellular carcinoma in liver. Panel 1 shows disorganized proliferation of neoplastic hepatocytes in sparse stroma with multifocal necroses (indicated by N). Panel 2 shows the neoplastic hepatocytes are highly pleomorphic with very high anisokaryosis (variation in nuclear size) and anisocytosis (variation in cell size), and a high mitotic rate with numerous bizarre mitotic figures (red asterisks). The neoplastic hepatocytes vary from very enlarged with a very large nucleus (black solid line) to more similar in size to normal hepatocytes (white solid line).

Bronchiolo-alveolar carcinoma in lung. Panel 3 shows poorly circumscribed, multinodular proliferation of neoplastic epithelial cells forming papillary, tubular and solid areas in sparse stroma (outlined in black; C: carcinoma; L: lung). Panel 4 shows the neoplastic epithelial cells are pleomorphic with numerous mitotic figures (red asterisks).

All histomicrographs are stained with hematoxylin-eosin. Scale bars: 250µm and 20µm as indicated.
