# Supplementary material for: Mice carrying nonsense mutant p53 develop frequent multicentric or metastatic tumors
Source: Cell Death Dis. 2025 Dec 11;17(1):85. doi: 10.1038/s41419-025-08290-9 (PMC12830816; doi:10.1038/s41419-025-08290-9)
Supplement: Supplementary file 10 — Supplementary Figure S6-S8 [file 41419_2025_8290_MOESM10_ESM.pdf]

Supplementary Figure S6

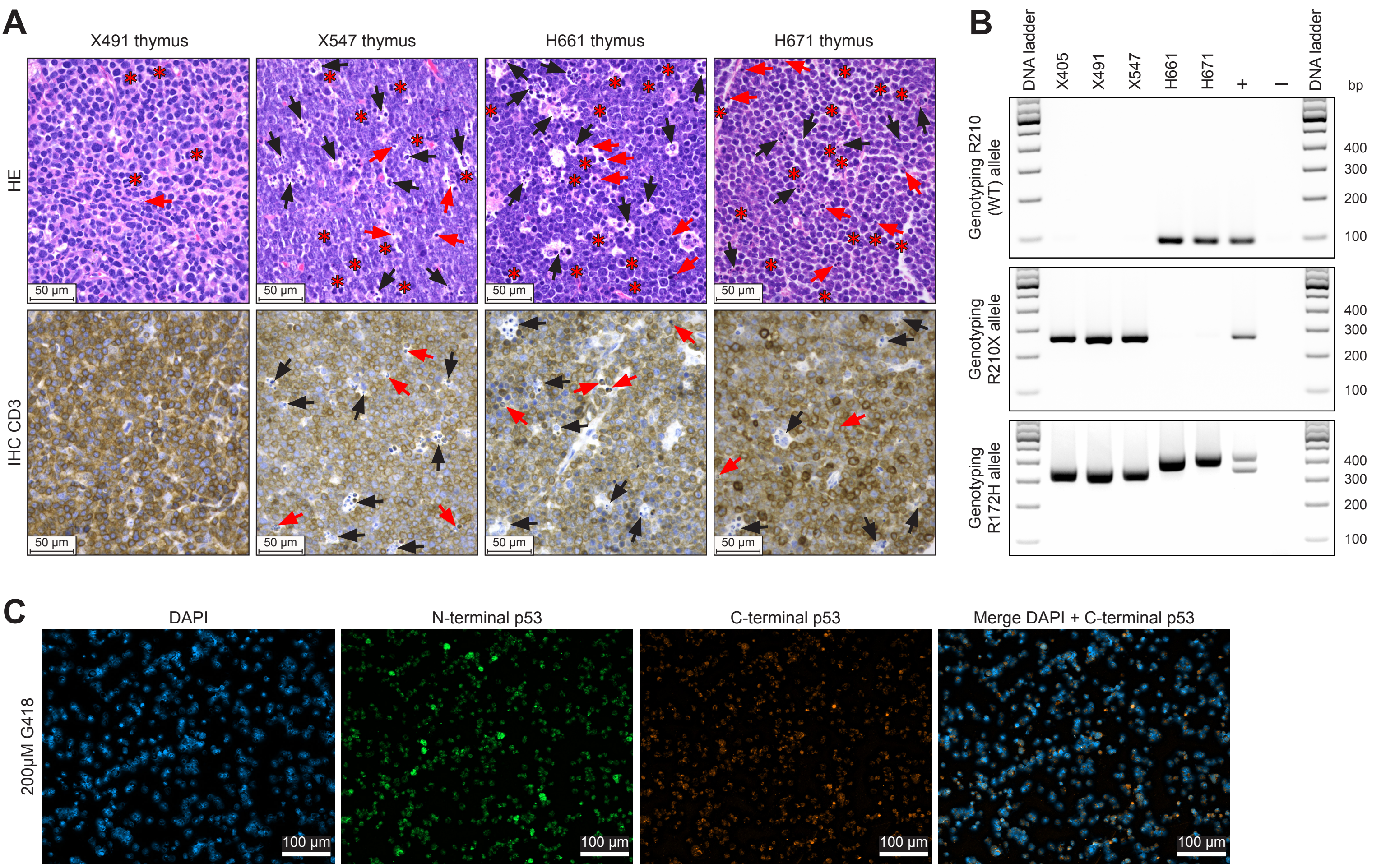

Supplementary Figure S6. Verification of mouse T-lymphoma cell lines and cell death induced by aminoglycoside G418, related to Figure 7.
(A) Representative histomicrographs of the thymus lymphomas from mice X491, X547, H661 and H671 stained with hematoxylin-eosin (HE, upper) and CD3 immunohistochemistry (IHC, lower). Mats of CD3-positive, i.e. T-cell origin, pleomorphic neoplastic lymphocytes with numerous mitoses (red asterisks), tingible body macrophages (black arrows), and cell fragments consistent with single-cell death (red arows) efface the normal thymic architecture in all thymi. **(B)** Verification of *Trp53*$^{R210X/R210X}$ (X405, X491 and X547) and *Trp53*$^{R172H/R172H}$ (H661 and H671) T-lymphoma cell line genotypes using the same PCR genotyping strategies as for mouse genotyping of the respective strain. All cell lines were genotyped using both protocols to ensure no cross-contamination has occurred. R210 (WT) allele is identified by a 95 bp PCR fragment (top panel) and R210X mutant allele by a 252 bp fragment (middle panel). Genotyping using the *Trp53*$^{R172H/R172H}$ strategy identifies WT allele by a 342 bp PCR fragment and R172H mutant allele by a 410 bp fragment (bottom panel) according to Lang *et. al.*, 2004 (9). All cell lines show clean and expected genotypes (X405, X491 and X547 = *Trp53*$^{R210X/R210X}$; and H661 and H671 = *Trp53*$^{R172H/R172H}$). **(C)** Representative immunofluorescence staining of X405 *Trp53*$^{R210X/R210X}$ T-lymphoma cells after 72h treatment with 200µM aminoglycoside G418. p53 was detected using N1 (N-terminal epitope) and 280aa C-term (C-terminal epitope) antibodies. Rightmost panel show merged DAPI and 280aa C-term p53 staining. This concentration of G418 caused substantial cell death, consistent with the Annexin V data shown in Fig. 7G. Scale bars: 50µm **(B)** and 100µm **(C)**.

Supplementary Figure S7

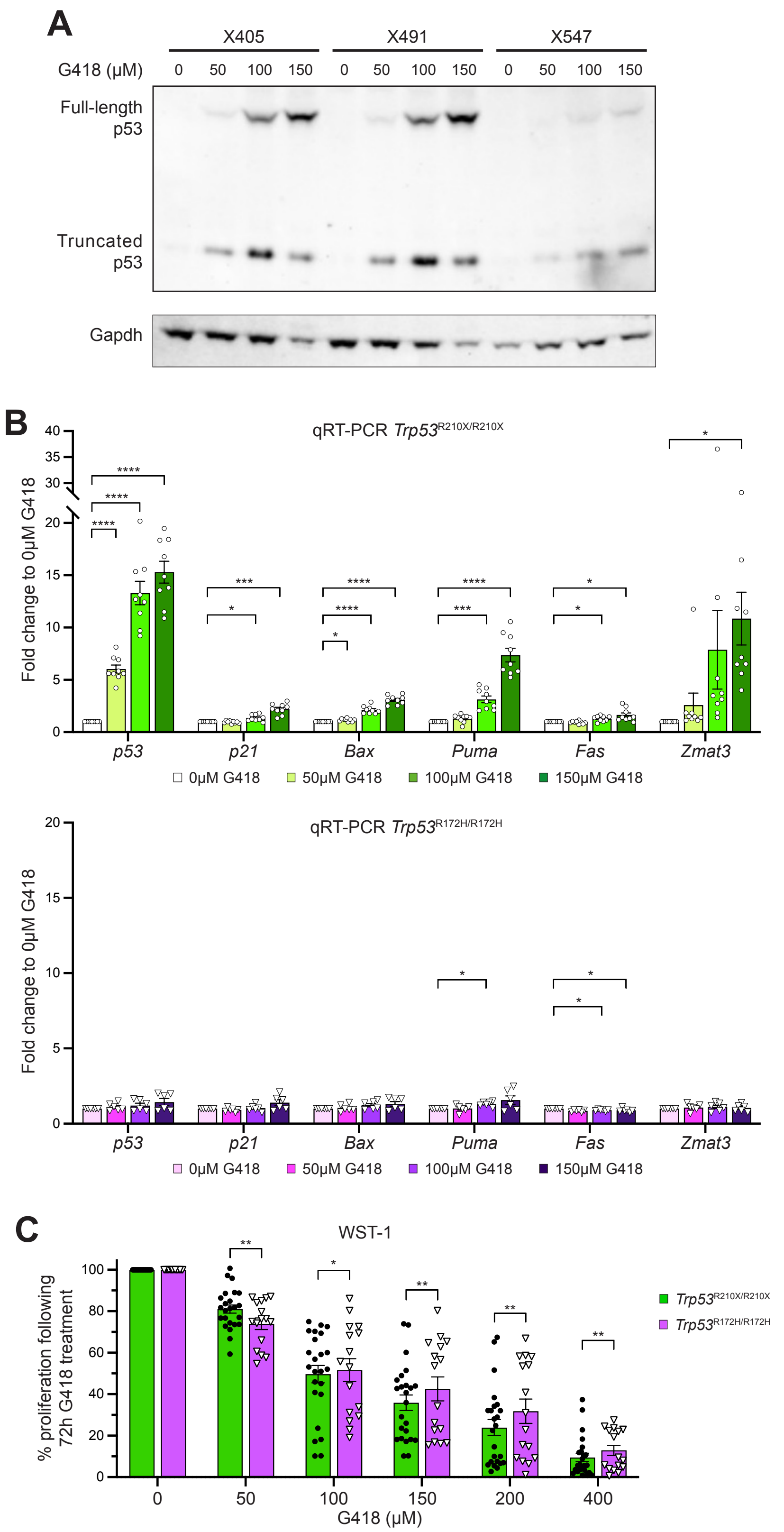

Supplementary Figure S7. Induction of full-length p53, upregulation of p53 target genes, and inhibition of cell proliferation in *Trp53*$^{R210X/R210X}$ T-lymphoma cells following G418 treatment, related to Figure 7.

(A) Western blot analysis showing dose-dependent induction of full-length p53 in three T-lymphoma cell lines following 72h treatment with G418 at indicated concentrations. p53 was visualized using the anti-p53 antibody 1C12 that recognizes an N-terminal epitope and therefore detects both full-length and C-terminally truncated p53. Gapdh was used as a loading control. **(B)** qRT-PCR results from Figure 7E showing all individual values from each cell line from 3 independent experiments per cell line. Gene expression values are normalized to *Gapdh* expression and compared to untreated (0µM G418) negative control for each gene. Upper panel: *Trp53*$^{R210X/R210X}$ T-lymphoma lines (n = 3); lower panel: *Trp53*$^{R172H/R172H}$ T-lymphoma lines (n = 2). **(C)** WST-1 assay results from Figure 7F showing all individual values from each cell line from 8 independent experiments per cell line. In **B** and **C**, statistical analysis was performed by repeated measures two-way ANOVA followed by Dunnett's multiple comparisons test **(B)**, and by two-way Mixed-effects analysis **(C)**, respectively. Mean ± SEM are indicated. Adjusted P-values: *$p < 0.05$, **$p < 0.01$, ***$p < 0.001$, ****$p < 0.0001$.

Supplementary Figure S8

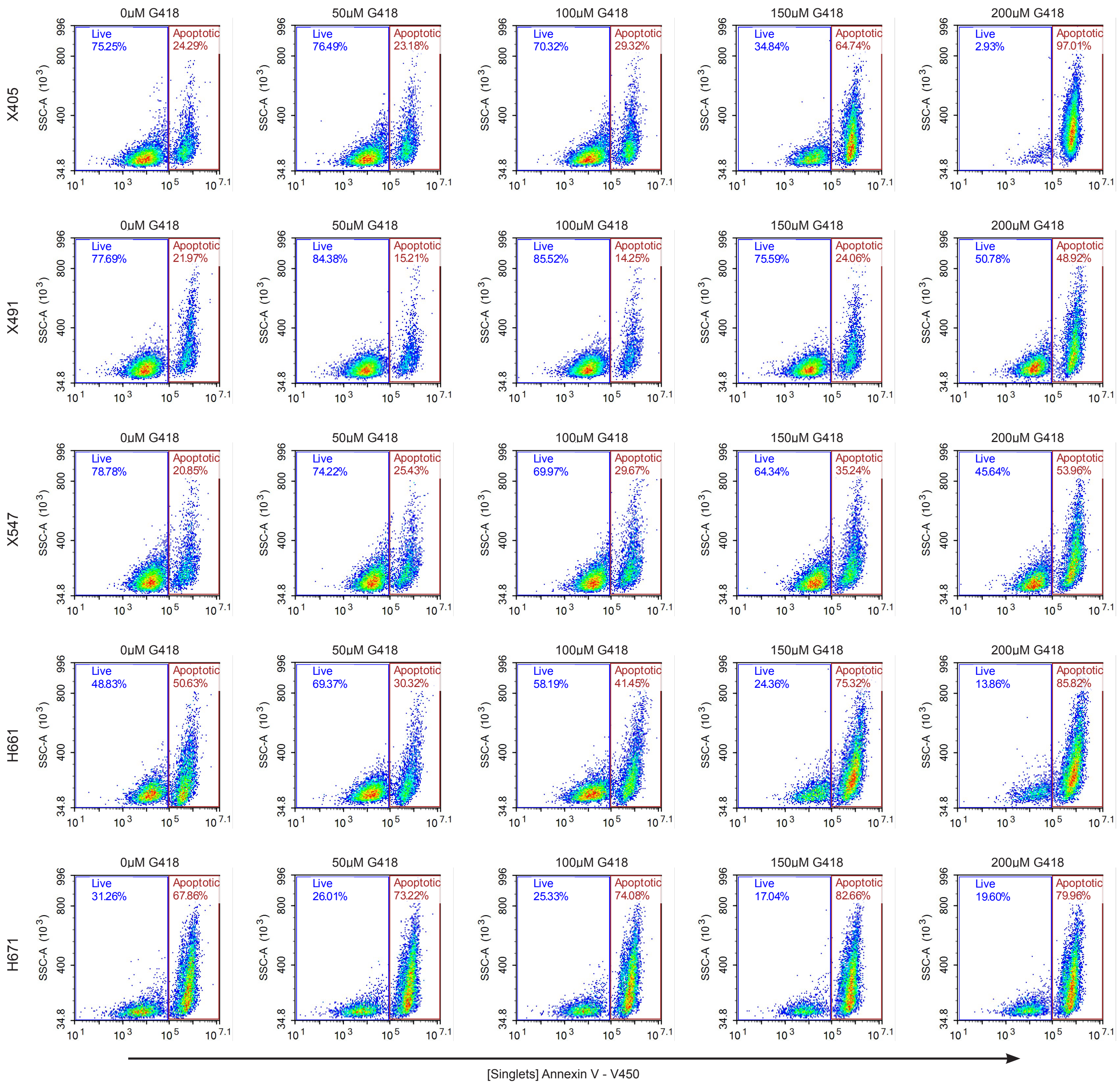

Supplementary Figure S8. Annexin V flow cytometry of *Trp53*$^{R210X/R210X}$ and *Trp53*$^{R172H/R172H}$ T-lymphoma cell lines following G418 treatment, related to Figure 7G.

Represenative plots from Annexin V flow cytometry of *Trp53*$^{R210X/R210X}$ (X405, X491 and X547) and *Trp53*$^{R172H/R172H}$ (H661 and H671) mouse T-lymphoma cell lines follwing treatment with G418 for 72h at indicated concentrations; 3 independent experiments per cell line were performed.
