# Supplementary material for: Mice carrying nonsense mutant p53 develop frequent multicentric or metastatic tumors
Source: Cell Death Dis. 2025 Dec 11;17(1):85. doi: 10.1038/s41419-025-08290-9 (PMC12830816; doi:10.1038/s41419-025-08290-9)
Supplement: Supplementary file 11 — Original Western Blots [file 41419_2025_8290_MOESM11_ESM.pdf]

Original Western blots, Figure 7D

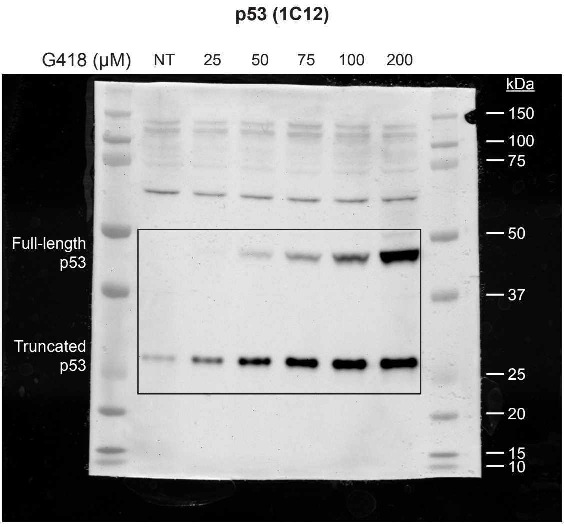

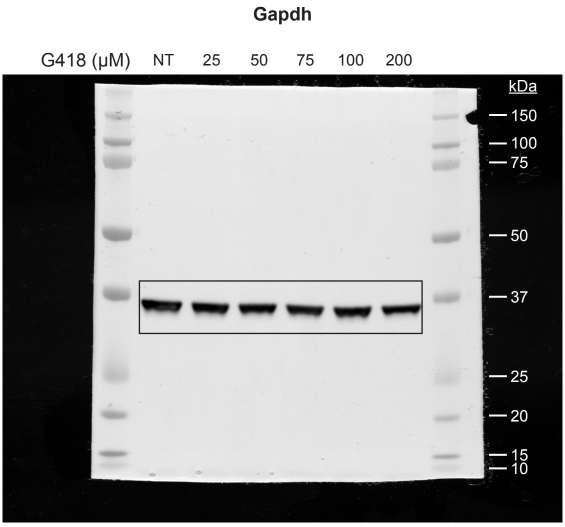

Original Western blots, Figure 7D.
The Western blot membrane shown in Fig. 7D was first blotted with the anti-p53 antibody 1C12 and an HRP-conjugated Rabbit-anti-Mouse IgG secondary antibody. 1C12 recognizes an N-terminal epitope and will therefore detect both full-length and C-terminally truncated p53. Following visualization of p53 and subsequent washing, the membrane was blotted with the HRP-conjugated G-9 anti-GAPDH antibody; Gapdh was used as a loading control. Squares indicate parts of the membrane shown in Fig. 7D. Molecular weight markers are shown.

Original Western blots, Supplementary Figure S7A

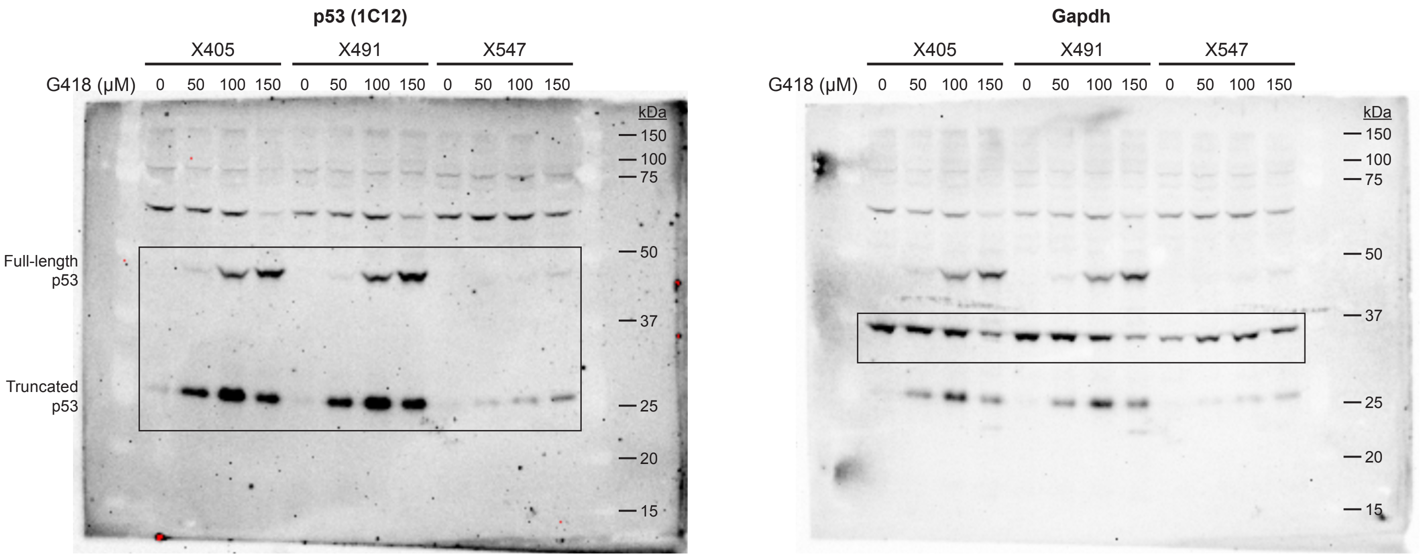

Original Western blots, Supplementary Figure S7A.
The Western blot membrane shown in Supplementary Fig. S7A was first blotted with the anti-p53 antibody 1C12 and an HRP-conjugated Rabbit-anti-Mouse IgG secondary antibody. 1C12 recognizes an N-terminal epitope and will therefore detect both full-length and C-terminally truncated p53. Following visualization of p53 and subsequent washing, the membrane was blotted with the HRP-conjugated G-9 anti-Gapdh antibody; Gapdh was used as a loading control. Squares indicate parts of the membrane shown in Supplementary Fig. S7A. Molecular weight markers are shown.
